# Place of Death Among Assisted Living Residents as a Factor of Hospice Regulations

**DOI:** 10.1093/geroni/igab046.2031

**Published:** 2021-12-17

**Authors:** Emmanuelle Belanger, Joan Teno, David Dosa, Wenhan Zhang, Pedro Gozalo, Kali Thomas

**Affiliations:** 1 Brown University School of Public Health, Providence, Rhode Island, United States; 2 Oregon Health and Science University, Portland, Oregon, United States; 3 Brown University, Providence, Rhode Island, United States; 4 Brown University, Brown University/Providence, Rhode Island, United States

## Abstract

Our objective was to examine the likelihood of dying in RC/AL among a national cohort of fee-for-service Medicare beneficiaries who died in 2018 (N=31,414) as a factor regulations allowing hospice care. We estimated multivariable logistic regression models to examine the association between RC/AL as place of death and supportive hospice regulations, controlling for demographic characteristics, dual Medicare/Medicaid eligibility, years in AL, and hospital referral region (HRR) to control for hospice practice patterns. A majority of beneficiaries in our cohort died in RC/AL; more than half while receiving hospice services. In unadjusted models, the odds of remaining in RC/AL communities until death were significantly higher in the presence of regulations supportive of hospice care. This relationship was no longer significant once adjusting for covariates and an HRR fixed effect, suggesting important variation in end-of-life experiences for AL residents not explained by hospice regulations.

